# Opioid substitution therapy in manipur and nagaland, north-east india: operational research in action

**DOI:** 10.1186/1477-7517-7-29

**Published:** 2010-12-01

**Authors:** Gregory Armstrong, Michelle Kermode, Charan Sharma, Biangtung Langkham, Nick Crofts

**Affiliations:** 1 Nossal Institute for Global Health, University of Melbourne, Victoria, Australia; 2 National AIDS Control Organisation - Guwahati Office, Ministry of Health & Family Welfare, Government of India; 3 Project ORCHID, Emmanuel Hospital Association, Guwahati, India

## Abstract

**Background:**

There is good evidence for the effectiveness of opioid substitution therapy (OST) for injecting drug users (IDUs) in middle and high-income countries but little evidence regarding the provision of OST by non-government organisations (NGOs) in resource-poor settings. This paper reports on outcomes of an NGO-based OST program providing sub-lingual buprenorphine to opiate dependent IDUs in two north-east Indian states (Manipur and Nagaland), a region where conflict, under-development and injecting of heroin and Spasmoproxyvon (SP) are ongoing problems. The objectives of the study were: 1) to calculate OST treatment retention, 2) to assess the impact on HIV risk behaviours and quality of life, and 3) to identify client characteristics associated with cessation of treatment due to relapse.

**Methods:**

This study involves analysis of data that were routinely and prospectively collected from all clients enrolled in an OST program in Manipur and Nagaland between May 2006 and December 2007 (n = 2569, 1853 in Manipur and 716 in Nagaland) using standardised questionnaires, and is best classified as operational research. The data were recorded at intake into the program, after three months, and at cessation. Outcome measures included HIV risk behaviours and quality of life indicators. Predictors of relapse were modelled using binary logistic regression.

**Results:**

Of all clients enrolled in OST during the month of May 2006 (n = 713), 72.8% remained on treatment after three months, and 63.3% after six months. Statistically significant (p = 0.05) improvements were observed in relation to needle sharing, unsafe sex, incidents of detention, and a range of quality of life measures. Greater spending on drugs at intake (OR 1.20), frequently missing doses (OR 8.82), and having heroin rather than SP as the most problematic drug (OR 1.95) were factors that increased the likelihood of relapse, and longer duration in treatment (OR 0.76) and regular family involvement in treatment (OR 0.20) reduced the likelihood of relapse.

**Conclusion:**

The findings from this operational research indicate that the provision of OST by NGOs in the severely constrained context of Manipur and Nagaland achieved outcomes that are internationally comparable, and highlights strategies for strengthening similar programs in this and other resource-poor settings.

## Background

Opioid substitution therapy (OST) is an evidence-based intervention for opiate dependant persons that replaces illicit drug use with medically prescribed, orally administered opiates such as buprenorphine and methadone. OST reduces HIV risk behaviours and harms associated with injecting (such as abscesses, septicaemia and endocarditis), overdose and participation in criminal activity, thereby improving the quality of life and health of injecting drug users (IDUs)[1-6]. It is endorsed by UNAIDS, UNODC and WHO as part of a comprehensive package of nine core interventions for IDU programs that collectively maximise impact for HIV prevention and treatment[7]. However, most of the evidence for OST effectiveness has been generated in middle and high-income countries where programs are mostly located in dedicated healthcare settings; evidence regarding the outcomes of OST programs in low-income countries where OST is often provided in grassroots settings such as drop-in-centres, is limited[3,8]. There are an estimated 106,000-223,000 IDUs in India, of whom only 5% are currently receiving OST, which is mostly delivered by community-based services[9,10]. There is a real need for evidence regarding outcomes of OST provision in India in order to strengthen the case for scaling up of services.

This paper reports on outcomes of an OST program providing buprenorphine to opiate dependent IDUs, delivered by non-government organisations (NGOs) in the north-east Indian states of Manipur and Nagaland. These states make up a region geographically isolated from the rest of India, and characterised by multiple sources of conflict including a longstanding civil insurgent struggle, poverty and unemployment. Approximately 2% of the population in Manipur and Nagaland inject drugs,[11] most commonly heroin and Spasmoproxyvon (SP, a synthetic opioid analgesic that contains dextropropoxyphene, dicyclomine hydrochloride and paracetamol). As a consequence, Manipur and Nagaland are the two states with the highest HIV prevalence in the country[11]. Both the epidemic and the response to it are more mature in Manipur, where sentinel surveillance data indicates that during the late 1990s HIV prevalence among IDUs approached 80%[12]. By 2007, HIV prevalence among IDUs was much reduced being 18% in Manipur and 1.9% in Nagaland[13]. The response to HIV and injecting drug use in this geo-politically complex environment was punitive and coercive, but harm reduction interventions such as needle and syringe exchange programs and condom distribution have been government policy since the mid 1990s[14].

Project ORCHID (Organised Response for Comprehensive HIV Interventions in the Districts of Nagaland and Manipur) is a Bill & Melinda Gates Foundation-funded HIV prevention project that has been working in selected districts of Manipur and Nagaland since 2004. It supports local partner NGOs to deliver a range of harm reduction interventions in rural and urban settings. In 2006, Project ORCHID initiated a buprenorphine-based OST program delivered by 11 local partner NGOs, initially with funding from the United Kingdom government's Department for International Developing (DFID), and subsequently from the National AIDS Control Organisation (NACO) and Emmanuel Hospital Association (EHA). The OST program is based in the community, operated out of drop-in centres. Sub-lingual buprenorphine is provided for registered IDUs seven days per week, and is administered by trained health care workers (mostly nurses) under the supervision of medical doctors, following a standardised protocol. The program was initially rapidly over-subscribed and waiting lists were created. The program is more fully described elsewhere[15].

During the DFID-funded period of the program (May 2006 - December 2007) more detailed information regarding characteristics of the clients and outcomes of the program were systematically collected as part of routine program monitoring. Analysis of these data were undertaken in order to address the following objectives: 1) to calculate OST treatment retention at 3, 6, 9 and 12 months, 2) to assess the impact of OST on HIV risk behaviours and quality of life, and 3) to identify client characteristics associated with reason for cessation of OST treatment.

## Methods

### Study design

This study involves analysis of data collected routinely during the implementation of an OST program, and is best classified as operational research, which can be defined as "The search for knowledge on interventions, strategies, or tools that can enhance the quality, effectiveness or coverage of programmes in which the research is being done" (p.711)[16]. There is a strong connection between program monitoring and evaluation and operational research. Study designs such as randomised controlled trials generate new knowledge about the efficacy of interventions in a controlled environment with strict inclusion and exclusion criteria, whereas operational research assesses effectiveness in routine settings that are far less controlled. The findings from operational research have direct and practical implications for health care delivery[16].

### Data collection

Data were prospectively collected from all clients enrolled in the OST program in Manipur and Nagaland between May 2006 and December 2007 (n = 2569, 1853 in Manipur and 716 in Nagaland) at intake, three months after entry into the program, and at cessation of treatment (regardless of the reason) using standardised questionnaires developed by the program. The questionnaires were interviewer-administered by the NGO nurse or outreach worker, and took approximately thirty minutes to complete. It was not always possible to conduct a face-to-face interview with clients at cessation of treatment, especially if cessation was due to relapse, so where necessary and possible, relevant information was drawn from the client file.

### Outcome measures

The intake and three month follow-up questionnaires captured self-reported information on socio-demographic characteristics, drug use, HIV risk behaviours, and quality of life. At cessation of treatment additional information was recorded regarding reason for cessation, family involvement during treatment, and adherence to treatment. Reasons for ceasing OST were categorised as: completed the program (meaning that the clients had withdrawn from buprenorphine and had not returned to their past pattern of drug use at the time of discharge); relapsed or involuntarily discharged (hereafter referred to as relapsed); and unknown reason for cessation.

### Analysis

Data were entered by the Project ORCHID monitoring and evaluation team using EpiInfo, and analysed using SPSS version 18. The statistical tests used were Chi-square, t-test, and McNemar's test, and statistical significance was calculated using two-tailed tests at the 95% confidence level. Clients who had ceased OST with an unknown reason (n = 281) were excluded from the analysis, except when calculating OST treatment retention and describing the client characteristics. In order to calculate OST treatment retention at 3, 6, 9 and 12 months, all clients commencing OST during May 2006 (n = 713) were tracked over the subsequent 12 months.

The impact of OST on HIV risk behaviours and quality of life was assessed by comparing changes between baseline and three month follow-up measures. Results were differentiated by the programmatic status of clients at the end of the data collection period i.e. completed the program, relapsed, or still on OST.

To determine factors associated with reason for cessation we identified all clients who had ceased treatment with a known reason for cessation (n = 895) i.e. those who had either completed the program or had relapsed. A binary logistic regression model was used to predict the likelihood of relapse at cessation of treatment rather than completion of the program. Unadjusted odds ratios with p-values less than 0.1 were considered eligible for the multivariate model, and gender and age were also included. The forced entry procedure was used to enter variables in the model.

## Results

### Client characteristics

Table 1 presents socio-demographic data for all clients at entry to OST disaggregated by state. In both Manipur and Nagaland, clients were predominantly male and the majority had at least a high school level of education. Almost half reported being unemployed and the most common source of referral to OST was friends/peers. A small proportion of the OST clients in Nagaland (13.2%) were female sex workers. Ages ranged from 16 to 61 years in Manipur with a mean age of 30.9 years. In Nagaland ages ranged from 18 to 55, with a mean age of 30.0 years.

**Table 1:** OST client socio-demographic characteristics at intake (n = 2569)*

Demographic characteristic	Manipur n (%)	Nagaland n (%)	Demographic characteristic	Manipur n (%)	Nagaland n (%)
**Sex**			**Education**		

Male	1775 (96.3)	598 (85.1)	No education	103 (5.6)	49 (6.9)

Female	69 (3.7)	105 (14.9)	Primary school	450 (24.3)	147 (20.6)

			High school	444 (24.0)	243 (34.1)

**Marital Status**			Undergraduate	548 (29.6)	146 (20.5)

Married	873 (47.1)	361 (50.5)	Graduate and above	306 (16.5)	128 (18.0)

Single	876 (47.3)	320 (44.8)			

Separated/divorced	67 (3.6)	23 (3.2)	**Occupation**		

Widowed	36 (1.9)	11 (1.5)	Unemployed	874 (48.6)	323 (45.3)

			Small business	335 (18.6)	72 (10.1)

**Source of referral**			Government	118 (6.6)	131 (18.4)

Friend/peer	950 (51.6)	370 (51.7)	Labourer	213 (11.8)	3 (0.4)

Outreach worker	280 (15.2)	148 (20.7)	Sex worker	1 (0.1)	94 (13.2)

Peer educator	287 (15.6)	95 (13.3)	Selling drugs	2 (0.1)	3 (0.4)

Family	207 (11.2)	71 (9.9)	Other	256 (14.2)	87 (12.2)

Nurse	6 (0.3)	1 (0.1)			

Other	111 (6.0)	30 (4.2)			

* Percentages were calculated excluding missing cases

There was variation in drug use between Manipur and Nagaland; at intake most clients in Manipur reported commonly using heroin (90.7%) whilst in Nagaland approximately equal proportions reported using heroin and SP (63.1% and 68.3% respectively). Clients from Nagaland more commonly reported use of other drugs including alcohol (50.9%), Relipen (20.4%; combination drug containing similar ingredients to SP) and Nitrosun (26.4%; nitrazepam). The majority of OST clients in Manipur identified heroin as their most problematic drug (87.6%), while in Nagaland the most problematic drug was evenly split between heroin and SP (50.2% and 47.7% respectively).

### OST treatment retention

Of all clients enrolled in OST during the month of May 2006 (n = 713), 72.8% remained on treatment after three months, and 63.3% after six months (Table 2). At the end of one year, 50.8% were still on OST. Approximately two-thirds (63.6%) had what can be defined as a positive outcome after one year i.e. 12.8% had completed the program and 50.8% were retained on treatment. Slightly more than one-quarter (27.5%) had ceased treatment at the end of one year due to relapse, and the remaining 9% had ceased treatment with an unknown outcome.

**Table 2:** OST treatment retention and outcomes over one year for a cohort of clients enrolled in May 2006 (n = 713)

	Retained on OST	Ceased - completed the program	Ceased - relapsed	Ceased - reason unknown
	n (%)	n (%)	n (%)	n (%)
**3 months**	519 (72.8)	18 (2.5)	138 (19.4)	38 (5.3)

**6 months**	451 (63.3)	42 (5.9)	166 (23.3)	54 (7.6)

**9 months**	405 (56.8)	60 (8.4)	186 (26.1)	62 (8.7)

**12 months**	362 (50.8)	91 (12.8)	196 (27.5)	64 (9.0)

### Impact of OST on HIV risk behaviours and quality of life

Substantial improvements in self-reported HIV risk behaviours were observed among clients retained on OST between intake and 3 months (Table 3). There were significant reductions in needle sharing and unsafe sex. At intake one-quarter of clients reported sharing needles in the past month compared to 2% or less after three months on OST. There was a significant decrease in the proportion of clients being jailed/detained. Reductions in HIV risk behaviours were observed amongst all clients on treatment, even those clients who went on to cease OST due to relapse.

**Table 3:** Changes in HIV risk behaviours when intake is compared with three months after enrolment (disaggregated by status of client at the end of the data collection period)

	Intake	3 months	p-value*
**Had shared a needle during past month (%)**			
Completed the program (n = 297)	23.5	0.7	<0.001
Relapsed (n = 155)	25.8	1.3	<0.001
Still on OST (n = 847)	27.6	2.1	<0.001
All clients (n = 1299)	26.5	1.8	<0.001

**Had an unsafe sexual encounter during past month (%)**			
Completed the program (n = 260)	14.6	9.6	0.11
Relapsed (n = 138)	15.4	4.3	0.01
Still on OST (n = 818)	15.5	7.6	<0.001
All clients (n = 1216)	15.3	7.6	<0.001

**Had been jailed/detained during past month (%)**			
Completed the program (n = 297)	10.8	0.3	<0.001
Relapsed (n = 155)	12.9	0.0	<0.001
Still on OST (n = 841)	11.7	1.1	<0.001
All clients (n = 1293)	11.6	0.8	<0.001

* McNemar's Test

There was a consistent and marked improvement observed in the quality of life measures when intake is compared with three months after enrolment (Table 4). Of the clients successfully followed-up at 3 months, the proportion reporting a good quality of life had risen by approximately 40-50%. Other statistically significant improvements in quality of life were also evident including increased attendance at social events, reduced frequency of family conflict, and a reduction in work-related absenteeism amongst those with a job. The improvements in quality of life were observed amongst all clients on treatment, even those clients who went on to cease OST due to relapse. Notably, no statistically significant changes were observed with respect to the proportion of clients who were employed.

**Table 4:** Changes in quality of life indicators when intake is compared with three months after enrolment (disaggregated by status of client at the end of the data collection period)

	Intake	3 months	p-value*
**Clients reporting a good quality of life (%)**			
Completed the program (n = 297)	14.5	65.7	<0.001
Relapsed (n = 155)	17.4	54.8	<0.001
Still on OST (n = 849)	13.0	63.5	<0.001
All clients (n = 1301)	13.8	63.0	<0.001

**Employed (%)**			
Completed the program (n = 297)	52.5	51.2	0.69
Relapsed (n = 155)	53.5	47.1	0.11
Still on OST (n = 844)	53.8	52.6	0.52
All clients (n = 1296)	53.5	51.6	0.17

**Days in family conflict during past month (mean)**			
Completed the program (n = 293)	4.6	0.5	<0.001^+^
Relapsed (n = 152)	4.1	0.8	<0.001^+^
Still on OST (n = 833)	4.6	0.7	<0.001^+^
All clients (n = 1278)	4.5	0.6	<0.001^+^

**Social events attended during past month (mean)**			
Completed the program (n = 297)	1.3	2.0	<0.001^+^
Relapsed (n = 155)	1.3	1.8	0.03^+^
Still on OST (n = 841)	1.3	2.0	<0.001^+^
All clients (n = 1293)	1.3	1.9	<0.001^+^

**Day absent from work during past month (mean)**			
Completed the program (n = 126)	2.5	0.6	<0.001^+^
Relapsed (n = 62)	1.9	1.0	0.22^+^
Still on OST (n = 350)	2.8	1.2	<0.001^+^
All clients (n = 538)	2.6	1.0	<0.001^+^

* McNemar's Test performed unless otherwise stated

+ Paired t-test

### Reasons for cessation of OST treatment

Of the 895 clients who ceased OST treatment during the data collection period, 57% (n = 510) left OST because they had relapsed, and 43% (n = 385) left because they had completed the program without a return to their previous pattern of drug use at the time of discharge.

Binary logistic regression modeling was performed to assess the relative impact of a range of factors on the reason for cessation (Table 5). The dependent variable was reason for cessation i.e. relapse versus completion of the program. The model contained gender and age as well as duration in treatment, most problematic drug, amount of money spent daily on drugs at intake, frequently missing more than two doses a week, and regular family involvement in treatment. This model explained between 43.9% (Cox and Snell R square) and 58.6% (Nagelkerke R square) of the variance in reason for cessation.

**Table 5:** Binary logistic regression model to predict the likelihood of relapse from OST treatment (n = 895)

Variable	Unadjusted Odds Ratio (95% C.I.)	p-value	Adjusted Odds Ratio (95% C.I.)	p-value
**Male**	1.22 (0.74, 2.02)	0.44	0.82 (0.34, 2.01)	0.67

**Age (years)**	1.01 (0.99, 1.04)	0.20	1.01 (0.97, 1.05)	0.63

**Duration in treatment (months)**	0.74 (0.71, 0.77)	<0.001	0.76 (0.72, 0.80)	<0.001

**Heroin as the most problematic drug (ref: SP)**	1.31 (0.96, 1.78)	0.09	1.95 (1.16, 3.28)	0.01

**Money spent daily on drugs at intake (Rs 100 units)**	1.18 (1.05, 1.33)	0.01	1.20 (1.00, 1.44)	0.05

**Frequently missed more than two doses a week**	14.67 (9.21, 23.35)	<0.001	8.82 (4.99, 15.63)	<0.001

**Regular family involvement in treatment**	0.11 (0.08, 0.15)	<0.001	0.20 (0.13, 0.30)	<0.001

Gender and age were not statistically significant predictors of reason for cessation. Five variables made a statistically significant contribution to the model; duration in treatment, most problematic drug, money spent daily on drugs at intake, frequently missing doses, and regular family involvement in treatment. Greater spending on drugs at intake, frequently missing doses, and having heroin rather than SP as the most problematic drug were factors that increased the likelihood of cessation due to relapse, and longer duration in treatment and regular family involvement in treatment reduced the likelihood of cessation due to relapse.

Among the clients who ceased treatment, those who reported heroin as their most problematic drug were almost twice as likely to relapse compared to those reporting SP. Clients who frequently missed more than two doses a week were almost nine times more likely to cease treatment due to relapse. Every additional month spent in treatment reduced the risk of cessation due to relapse by 24%. Clients whose families were not regularly involved in their OST treatment were five times more likely to cease treatment due to relapse.

## Discussion

This study aims to contribute to the evidence-base for the provision of OST by NGOs in northeast India, a complex setting where injecting drug use and a consequent HIV epidemic present substantial public health challenges. Previous studies have highlighted the positive impact of OST on reducing HIV risk behaviours, and improving the quality of life and health of IDUs, and the findings of this study also support the efficacy of OST as an intervention for people with opioid drug dependence.

### HIV risk behaviours and socio-economic outcomes

A range of behavioural, social and economic benefits were evident as early as three months into treatment, and many of these benefits extended beyond the OST clients to their families and communities. An important outcome of the OST program was a substantial reduction in reported HIV risk behaviours, primarily unsafe injecting and unsafe sex. Participation in the program arguably reduced the risk of HIV transmission not only for those attending the program but also for their sexual partners (often wives) and children. Other studies have also found that OST is associated with rapid reductions in HIV risk behaviours[3,5,6,17].

Less family conflict has multiple positive flow-on effects, especially for children. Fewer episodes of detention or imprisonment reduces exposure to HIV risks, and suggests that less crime is being committed, an important social outcome. One potential benefit of OST programs not evident in this study is an increase in employment for the clients. This may be due to the fact that meaningful employment opportunities for relatively well-educated young people in north-east India are extremely limited. Additionally, many (male) drug users in north-east India are cared for by their natal families, so are not forced to do menial work in order to obtain the basic necessities of life.

### Retention in treatment

Retention in OST treatment in Manipur and Nagaland (63% after six months) is comparable with retention outcomes reported by a WHO collaborative study that included sites from low, middle and high-income countries (approximately 70% after six months overall - only 55% in Australia)[3]. Retention in treatment is clearly important for the success of OST programs. As the findings from this research and other studies indicate, the longer people are retained in an OST program, the greater the likelihood that they will complete the program rather than relapse[17].

Other studies have reported buprenorphine dose as an important determinant of retention in the treatment program (higher doses being associated with better retention) [1]. While information about the dose of buprenorphine at the point of cessation was recorded for some of the clients in this study, the extent of missing data for this variable precluded meaningful analysis.

### Implications for policies and programs

Almost half of the clients who ceased OST did so having completed the program without returning to their previous pattern of opiate drug use at the time of discharge, whilst the other half ceased OST due to relapse. Factors that significantly increased the likelihood of ceasing treatment due to relapse were higher spending on drugs at intake, frequently missing buprenorphine doses, and reporting heroin as their most problematic drug. Longer duration in treatment and regular family involvement significantly decreased the likelihood of relapse.

Strategies to strengthen retention rates need to be identified and implemented in order to achieve successful outcomes for a larger proportion of clients. Facilitating family involvement in OST treatment could help to achieve better outcomes for clients, as might active follow-up and additional support for clients who are regularly missing buprenorphine doses and for those who identified heroin (rather than SP) as their most problematic drug. Additionally, given that a large proportion of clients who relapse leave the program in the first few weeks, more intensive support for clients over the first months of treatment may be beneficial.

Clients classified as "completing the program" were not necessarily totally abstinent, but were no longer requiring buprenorphine and had not returned to their former pattern of drug use at the time of discharge. Substantial reductions in drug use and HIV risk behaviours should be the goal of OST, rather than abstinence[17]. A systematic review of published research from 1966 to 2003 reported that post-treatment abstinence rates varied between 22% and 86%; overall 33% of former OST (methadone) patients were abstinent from at least opioids for an average of more than two years after completing detoxification[18]. Another review of OST research conducted in Germany (methadone) reported that only 10% of clients became totally abstinent, and identified the concern that attempts to introduce time-limited (abstinence-oriented) treatment would result in relapse and physical and psychological instability[19]. It is probable that the situation is similar in north-east India i.e. only a small proportion of OST clients are likely to achieve long-term total abstinence. Many will need to remain in the program for years, and some will require lifelong treatment.

### Limitations

An important limitation of this analysis is that the data were based primarily on self-report measures. Social acceptability bias may have influenced the IDUs to understate the extent to which they were engaging in HIV risk behaviours, particularly given that the data were being collected by program staff, though the comparability of the results with controlled trials suggests that any effect of this kind may have been minimal. The OST program in north-east India was better resourced when funded by DFID than is currently the case (OST is now funded by government), so we cannot assume that current outcomes are the same as those reported in this study.

The length of follow-up is too short to draw any firm conclusions about longer-term outcomes for these OST clients. A prospective longitudinal cohort study to systematically follow OST clients for 1-2 years would provide valuable information about outcomes for OST clients, the impact of various dosing schedules, social and economic benefits, program costs, and the extent to which clients are cycling in and out of the program. Additionally, it would be useful to follow clients who cease treatment (for whatever reason) to compare the benefits of staying in treatment over those of leaving. A qualitative investigation to follow-up clients who relapse, in order to better understand their reasons for relapse, would contribute to more effective programming.

The findings from this study indicate that this OST program in Manipur and Nagaland, which was implemented by NGOs in a severely constrained context managed to achieve outcomes that are internationally comparable. It has arguably made an important contribution to HIV prevention in the region, as well as improving the quality of life for a large group of people with opioid dependence, their families and communities.

## Competing interests

The authors declare that they have no competing interests.

## Authors' contributions

All authors contributed to interpretation of the findings and development of the manuscript. GA and MK undertook the statistical analysis and wrote the first draft of the manuscript. BL and CS provided expertise on the context of local program delivery. NC provided expertise on opioid substitution therapy and harm reduction. All authors read and approved the final manuscript.

## Authors information

CS was the OST Project Coordinator for Manipur and Nagaland during the collection of data.
